# The genetic basis of multiple system atrophy

**DOI:** 10.1186/s12967-023-03905-1

**Published:** 2023-02-10

**Authors:** Fan Shuen Tseng, Joel Qi Xuan Foo, Aaron Shengting Mai, Eng-King Tan

**Affiliations:** 1 Division of Medicine, Singapore General Hospital, Singapore, Singapore; 2 Department of Neurosurgery, National Neuroscience Institute, Singapore, Singapore; 3 Yong Loo Lin School of Medicine, National University of Singapore, Singapore, Singapore; 4 Department of Neurology, National Neuroscience Institute, Singapore, 169856 Singapore; 5 Duke-NUS Medical School, Singapore, Singapore

**Keywords:** Multiple system atrophy, Neurodegeneration, Movement disorders, Genetics, Mutations, Polymorphisms

## Abstract

Multiple system atrophy (MSA) is a heterogenous, uniformly fatal neurodegenerative ɑ-synucleinopathy. Patients present with varying degrees of dysautonomia, parkinsonism, cerebellar dysfunction, and corticospinal degeneration. The underlying pathophysiology is postulated to arise from aberrant ɑ-synuclein deposition, mitochondrial dysfunction, oxidative stress and neuroinflammation. Although MSA is regarded as a primarily sporadic disease, there is a possible genetic component that is poorly understood. This review summarizes current literature on genetic risk factors and potential pathogenic genes and loci linked to both sporadic and familial MSA, and underlines the biological mechanisms that support the role of genetics in MSA. We discuss a broad range of genes that have been associated with MSA including genes related to Parkinson’s disease (PD), oxidative stress, inflammation, and tandem gene repeat expansions, among several others. Furthermore, we highlight various genetic polymorphisms that modulate MSA risk, including complex gene–gene and gene-environment interactions, which influence the disease phenotype and have clinical significance in both presentation and prognosis. Deciphering the exact mechanism of how MSA can result from genetic aberrations in both experimental and clinical models will facilitate the identification of novel pathophysiologic clues, and pave the way for translational research into the development of disease-modifying therapeutic targets.

## Introduction

Multiple system atrophy (MSA) comprises a group of clinically heterogenous, uniformly fatal, progressive neurodegenerative conditions associated with dysautonomia, parkinsonism, cerebellar dysfunction and corticospinal degeneration [1–4]. MSA is broadly categorized into the Cerebellar subtype (MSA-C) and Parkinsonism subtype (MSA-P), depending on the predominant neurological presentation [5]. Recently, the International Parkinson and Movement Disorder Society (MDS) revised the diagnostic criteria for MSA using an evidence-based and consensus-based approach [6]. These criteria classify MSA into four groups with varying diagnostic certainty: neuropathologically established MSA, clinically established MSA, clinically probable MSA, and possible prodromal MSA.

MSA is regarded as an ɑ-synucleinopathy, with its neuropathological hallmark being glial cytoplasmic inclusions (GCI) in oligodendrocytes [7–9]. The exact pathogenesis is poorly understood, but has been postulated to arise from ɑ-synuclein overexpression and accelerated uptake in neurons and oligodendrocytes, impaired ɑ-synuclein degradation from autophagic and proteasomal dysfunction, mitochondrial dysfunction, oxidative stress, and neuroinflammation [10].

**Fig. 1:**
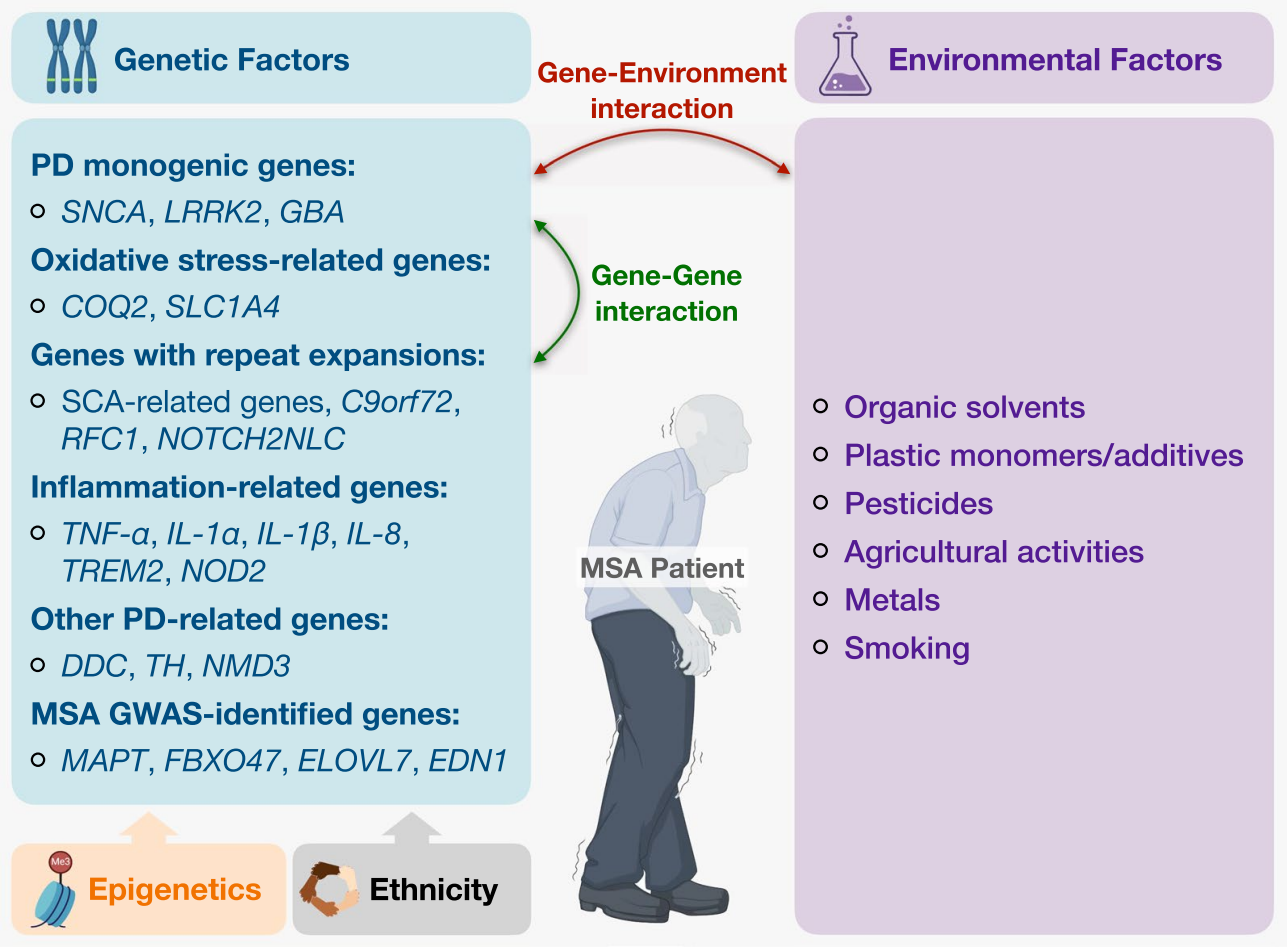
Both genetic and environmental factors influence MSA risk. Genetic factors, whose expression is influenced by epigenetics and ethnicity, include genes associated with monogenic forms of PD, genes related to oxidative stress, genes with repeat expansions, genes related to inflammation, other genes related to PD and genes identified through MSA GWAS. The pathophysiology is further complicated by complex gene–gene and gene-environment interactions that have yet to be fully elucidated. *GWAS* genome-wide association study, *MSA* Multiple System Atrophy, *PD* Parkinson’s Disease

MSA is widely regarded as a primarily sporadic disease, with a possible genetic component (Fig. 1). Familial forms are rare, with pooled estimates of heritability approximated to be 2.09–6.65% [11] alongside case reports of multiplex families with both autosomal dominant and autosomal recessive inheritance patterns [12–16].

Current knowledge of the genetics of MSA is limited. To address this gap, we provide a concise review of published literature on genetic risk factors and potential pathogenic genes and loci linked to both sporadic and familial MSA, and outline the biological basis and evidence that support the genetic underpinnings in its pathophysiology. Furthermore, we highlight complex gene–gene and gene-environment interactions which influence the disease phenotype and have clinical significance in both presentation and prognosis.

### SNCA

The *SNCA* gene (ɑ-synuclein, 4q22.1) encodes ɑ-synuclein, a protein that is found mainly in the presynaptic terminals of neurons and contributes to synaptic transmission [17, 18]. Similar to Parkinson’s disease (PD), *SNCA* has been of great interest since MSA is classified as an ɑ-synucleinopathy and GCI mainly contains filamentous, insoluble ɑ-synuclein. To date, no pathogenic *SNCA* mutation have been associated with monogenic forms of MSA. Although several case studies have reported rare mutations (including G51D, A53E), they have not been replicated in larger cohorts [19–21].

Studies looking at *SNCA* SNPs have been more promising. Scholz et al. [22] identified *SNCA* variants rs11931074 and rs3857059 to be significantly associated with MSA in a European population, with the former association also observed by Ross et al. [23]. A separate European study found another two *SNCA* variants that were linked to MSA, rs3822086 and rs3775444 [24]. However, these findings could not be replicated in Asian populations [25–27] and this could, in part, be related to the differences in the frequency of the risk alleles in different populations since the prevalence of the rs11931074 “T” allele is considerably higher in Asian populations (51–58%) than European populations (2–10%) [25]. All Asian studies also recruited clinically-diagnosed MSA patients only, compared to the European studies which included pathologically-diagnosed MSA patients. Other SNCA variants, including specific SNPs linked to PD (rs2736990 and rs356220) and a set of tagging SNPs estimated to represent 95% of haplotype diversity have not been shown to modify MSA risk [28–30].

*SNCA* copy number variations (CNV) have been associated with MSA, with copy number gains and resulting increase in *SNCA* expression leading to greater ɑ-synuclein inclusions in both the non-neuronal and neuronal cells of MSA subjects [31–33]. This was correlated with earlier onset of disease, reflecting the clinical implications of gene dosage on disease presentation. An earlier study of 58 MSA cases did not observe any *SNCA* gene multiplication, but there were limitations in the methodology as only whole gene multiplication was evaluated [34]. Larger cohorts are needed to draw relations between *SNCA* CNVs and MSA.

Of note, a genome-wide association study conducted in patients with MSA of European ancestry failed to detect any association between *SNCA* and MSA [35]. This may be attributed to interpopulation heterogeneity of *SNCA*, as observed by the authors.

### LRRK2

*LRRK2* (leucine-rich repeat kinase, 12q12), also referred to as dardarin or PARK8, is a large protein that has both kinase and GTPase activity [36]. Mutations are associated with autosomal dominant and sporadic late-onset PD, with incomplete and age-variable penetrance [37–39]. As pathological studies have revealed significant pleomorphism at the cellular level (including Lewy bodies and tau/ubiquitin inclusions) [40], various groups have investigated the association of *LRRK2* mutations and other neurodegenerative conditions.

*LRRK2* G2019S is the most commonly occurring pathogenic mutation, especially among the Ashkenazi Jewish, North African Arab and Spanish populations [41–43]. Studies thus far have failed to establish an association between *LRRK2* G2019S and MSA [44–46], although interestingly a recent case report detected the mutation in a Caucasian subject who had pathologically-diagnosed MSA [47].

In a large combined US-UK series, *LRRK2* M2397T polymorphism was protective for MSA, with a stronger association observed in the US cohort and for MSA-P/MSA-mixed patients [48]. A similar negative correlation was observed for G1624G, M1646T and N2081D within the US group, and N551K and R1398H within the UK group, but observed associations did not reach statistical significance.

Other *LRRK2* variants (R1628P, G2385R) have also been investigated but no association has been found [49–52], with the exception of a case report of a rare variant Ile1371Val in an MSA patient [53].

### GBA

*GBA* (glucocerebrosidase, 1q21) homozygous mutations are associated with Gaucher Disease, and more than two hundred pathogenic variants have been identified [54]. Pathogenic *GBA* variants have been demonstrated to increase the risk of developing PD [55–59] and dementia with Lewy bodies [60, 61]. A large-scale multicenter study identified twenty heterozygous *GBA* SNPs amongst MSA patients, of which nine are known to be pathogenic for Gaucher Disease (R120W, G202R, F213I, N370S, G377S, D409H, L444P, L444R, RecNciI) [62]. The pooled results across the North American, European, and Japanese series were statistically significant, but only the North American cohort reached significance when analyzed separately. One possible explanation could be the relatively large proportion of Ashkenazi Jews in North America compared to other parts of the world (with the exception of Israel), given that Gaucher Disease (especially type 1) has higher incidence in the Ashkenazi Jewish population compared to other ethnicities [63]. This was further supported by US studies which found significant associations between *GBA* SNPs and MSA, with one study noting that 3 out of the 6 Ashkenazi Jews in the study carried *GBA* mutations [64, 65]. Comparatively, there was no significant relationship between disease-causing *GBA* variants and MSA in European and Asian populations [66–70]. Functional studies suggest that lysosomal dysfunction as a result of *GBA* deficiency dysregulates ɑ-synuclein processing and induces its aggregation [71, 72]. This relationship is further complicated by other molecular regulatory mechanisms, such as the Thyroid Hormone Receptor Interacting Protein 12 (TRIP12), which ubiquinates glucocerebrosidase and influences GBA expression [73].

### COQ2 and other oxidative stress-related genes

*COQ2* (coenzyme Q2, polyprenyltransferase, 4q21.23) encodes an important enzyme in the Coenzyme Q10 (CoQ) biosynthetic pathway, with loss-of-function mutations resulting in CoQ deficiency and consequent increase in mitochondrial oxidative stress with reduction in ATP synthesis [74, 75]. Reduction in *COQ2* expression with corresponding decrease in CoQ and ATP levels have been shown in both the brain tissue and plasma of MSA patients, implicating CoQ biosynthesis in the pathogenesis of MSA [76–78].

The Multiple-System Atrophy Research Collaboration (MSARC) first published findings of a possible association between *COQ2* and MSA after identifying a homozygous mutation (M128V-V393A/M128V-V393A) and compound heterozygous mutations (R387X/V393A) in COQ2 in two multiplex Japanese families [79]. The allele frequency of the V393A variant was found to be higher in MSA patients than controls within the Japanese series (4.8% vs 1.6%), but the variant was not found in any of the MSA patients or healthy controls in the European or North American series. In addition, this observation was made mainly within the MSA-C subgroup. Although the results were not always reproducible [80–84], other East Asia population case-control studies and meta-analyses showed a significant association between the V393A variant and MSA-C patients [85–87], suggesting that this genetic susceptibility is, at least in part, specific to certain populations and ethnicities [88–92]. The rarity of V393A polymorphism in the Caucasian population may also explain the lack of replicability.

Several other genetic polymorphisms have been reported (S107T, M128R, M128V, R387X, R197H, S146N, L402F, R173H, A32A, L25V, N386I, L162F), however larger sample sizes are needed to confirm their association [80–83, 85, 90].

Other genes involved in oxidative stress have been evaluated. Soma et al. [93] examined eight genes (*CHOP*, *ATF3*, *CEBPB*, *SQSTM1*, *CARS*, *SLC1A4*, *ATF4*, *EIF4EBP1*) involved in oxidative stress pathways and found significant associations between *SLC1A4* rs759458 and MSA. Secondary analysis further uncovered several haplotypes of *SLC1A4*, *SQSTM1* and *EIF4EBP1* that altered MSA risk. Oxidative stress involves multiple complex pathways with various gene–gene interactions, thus requires more studies to further elucidate these mechanisms.

### MAPT

The *MAPT* gene (microtubule associated protein tau, 17q21.31) encodes tau, a protein which confers and maintains neuronal microtubule stability, and whose aberrant deposition in neuronal or glial cells results in neurodegenerative disorders known as tauopathies [94–99]. There are 2 extended *MAPT* haplotypes H1 and H2, with H1 further divided into subhaplotypes (e.g., H1c, H1b, etc.) [100]. *MAPT* has been shown to affect susceptibility to PD [101], Alzheimer’s Disease [102–104], frontotemporal dementia [105], progressive supranuclear palsy [106], corticobasal degeneration [107] and dementia with Lewy bodies [108].

An association between H1 haplotype (rs1052553) “A” allele and MSA has been reported by some investigators, but not for the H1c subhaplotype (rs242557) [109]. The same group conducted a follow-up study [110] using a larger sample size and six tagging SNPs to capture > 95% of the haplotype diversity and define over twenty H1 haplotypes [111, 112]. When analyzing individual SNPs, three variants (rs242557, rs3785883 and rs8070723) modulated MSA risk amongst pathologically-diagnosed patients. In the haplotype analysis, two risk haplotypes (H1x and H1J) were identified amongst pathologically-diagnosed patients, although this differed from the one (H1U) identified amongst clinically-diagnosed patients. Separately, two protective haplotypes (H2 and H1E) were identified, with the H2 haplotype showing a significant association for MSA-C and MSA-mixed subtypes only.

Chen et al. studied *MAPT* rs242557 in a Chinese population but did not find any relation with MSA, which could be attributed to ethnic differences or diagnostic inaccuracies since this study included clinically-diagnosed patients only.

### SCA-related genes, C9orf72, and other repeat expansions

Spinocerebellar Ataxia (SCA) refers to a broad group of genetic disorders where cerebellar ataxia is a common feature, with CAG repeat expansions as the most common underlying genetic anomaly [113, 114]. Trinucleotide repeat expansions in SCA-implicated genes have been shown to increase the risk of amyotrophic lateral sclerosis and depression [115–119], and affect disease severity in Alzheimer’s disease [120].

Studies have identified intermediate and pathologic expansions in SCA-related genes in MSA patients. This seems to vary between ethnicities as *ATXN1* (Ataxin 1) and *ATXN2* (Ataxin 2) (corresponding to SCA-1 and SCA-2 respectively) were implicated in an Italian population [121], whereas the majority of patients in a Korean population had repeat expansions in *TBP* (TATA-box binding protein) (corresponding to SCA-17) [122]. It is unclear if the larger number of CAG repeats in normal alleles of *ATXN1* and *ATXN2* amongst Caucasians is a contributory factor [123]. These expansions seem to be more associated with MSA-C than MSA-P, but more data are still needed as cases have been reported in both groups [124]. Interestingly, studies have also shown a higher mean CAG repeat length in MSA patients compared to controls [121, 125, 126].

Caution is needed in drawing definitive conclusions as all the included studies relied primarily on a clinical diagnosis of MSA based on consensus criteria, which may lead to diagnostic inaccuracies since both conditions can present with cerebellar dysfunction and parkinsonian features [127, 128]. This raises the possibility of misdiagnosis [129, 130] or dual pathologies [131, 132] rather than an underlying genetic association.

The hexanucleotide GGGGCC repeat expansion in *C9orf72* (chromosome 9 open reading frame 72, 9p21.2) is most commonly associated with amyotrophic lateral sclerosis and frontotemporal dementia [133–135], but has also been detected in rare cases of PD, Alzheimer’s Disease, psychosis and atypical parkinsonism [136–143]. Goldman et al. were one of the first to report a link between *C9orf72* repeat expansion and MSA in a pair of siblings carrying the mutation, and who were each diagnosed with clinical MSA and ALS respectively [144]. Subsequent publications, which comprised Caucasian and Asian cohorts and included pathologically-diagnosed MSA patients, could not replicated the findings [145–149]. However, a recently published Italian study found heterozygous mutations in the pathological range in two patients and intermediate/premutation range in four patients [150]. Given the small sample size (n = 100) and lack of neuropathological diagnosis of this study, more work needs to be done to further elucidate the role of *C9orf72* repeat expansions in MSA.

*RFC1* (Replication Factor C Subunit 1) biallelic intronic repeat expansions is associated with cerebellar ataxia, neuropathy and vestibular areflexia syndrome (CANVAS) [151]. Given the similar clinical presentation with MSA-C, pentanucleotide repeat polymorphisms of *RFC1* were investigated in recent studies. Wan et al. found biallelic and heterozygous AAGGG repeat expansions in three and thirteen clinically-diagnosed MSA patients respectively, but the association did not reach statistical significance [152]. Other studies, including one consisting exclusively of pathologically-diagnosed MSA patients only, did not identify any *RFC1* repeat expansions [153, 154].

Trinucleotide repeats in *NOTCH2NLC* is responsible for neuronal intranuclear inclusion disease (NIID) [155]. Pathogenic GGC repeat expansions (at least 100 repeats) were identified in 2.6% of clinically-diagnosed MSA patients in one study [156], but were not present in any of the MSA patients in another study [157]. In the former, the patients with GGC repeat expansions had longer disease duration, slower progression and ɑ-synuclein-negative skin biopsies, suggesting a non-MSA condition or an underlying dual pathology.

### Inflammation-related genes

Neuroinflammation has been a purported mechanism in the pathogenesis of MSA [158–160]. The consequent microglial activation, cytokine and chemokine release, and pro-inflammatory conditions are thought to accelerate ɑ-synuclein aggregation and oligodendroglial apoptosis [161, 162].

Genes encoding various interleukins (IL), TNF-ɑ and other inflammatory mediators have thus been an area of interest. The high producer allele “C” of gene polymorphism TNF-ɑ-1031C/T (rs1799964) was found to increase risk of MSA in both genotype distribution and minor allele frequency [163, 164]. IL-1ɑ-889 (rs1800587) allele “T”-carrying genotypes, associated with higher transcriptional activity, were also overrepresented in MSA with a positive gene dose effect in Caucasians [165]. However, this was not observed in two separate Asian series [164, 166].

The IL-8-251 (rs4073) “T” allele was found to increase risk of MSA in a dose-dependent manner despite having lower transcriptional activity than the “A” allele, a relationship that strengthened in individuals who also carry the intercellular adhesion molecule-1 (ICAM-1: E469K) “KK” genotype [167]. IL-1β-511 (rs16944) low producer allele “A” was also noted in greater frequency amongst MSA patients compared to allele “G” [166], and even contributed to earlier onset of disease [164]. This underscores the complexity of neuroinflammatory responses, especially since some cytokines play critical roles in neuronal regeneration and can confer early protection against neurodegeneration [168, 169].

*TREM2* (Triggering Receptor Expressed On Myeloid Cells 2) encodes a receptor that binds TYROBP (TYRO protein tyrosine kinase-binding protein) and forms a signaling complex that is involved in microglial activation, neuroinflammation and cytokine production [170]. *TREM2* variants, specifically rs75932628 (p.R47H), have been implicated in neurodegenerative disorders such as PD [171], Alzheimer’s Disease [172, 173], amyotrophic lateral sclerosis [174] and frontotemporal lobe dementia [175]. This substitution was shown to be associated with increased risk of MSA in a Caucasian population, although this relationship weakened after adjusting for age and sex [176]. It is possible that this loss-of-function mutation affects myelin homeostasis and reduces clearance of myelin debris, causing microglial activation. Although a separate study in a Chinese population only found one patient carrying the T allele [177], this may be due to the relative rarity of this polymorphism amongst Asians [178].

Shadrin et al. recently employed a genome-wide genetic pleiotropy-informed approach to investigate the link between MSA and seven autoimmune diseases [179], and found substantial polygenic overlap between inflammatory bowel disease and MSA with three shared genetic loci (rs4957144 in the first intron of *C7*, rs12740041 and rs116843836 upstream of *DENND1B* and *RSPO4* respectively). A transgenic mice model further showed that *C7* expression in the midbrain was dysregulated. The effects for rs4957144 and rs12740041 on MSA and IBD were in opposite directionality, suggesting that these shared genes likely have complicated and differing pathogenic mechanisms on these diseases. It further lends credibility to the gut-brain axis theory and a connection between chronic bowel immune dysfunction and neuroinflammation [180–182].

The rs3135500 variant in the *NOD2* gene, which activates nuclear factor κB (NK-κB) mediated inflammation, was shown to increase risk of MSA and correlate with increased peripheral mononuclear cell mRNA NO2 and plasma NOD2 protein levels [183, 184]. Acute phase reactant alpha 1-antichymotrypsin (ACT), encoded by the *SERPINA3* gene, was also found in higher levels in the cerebrospinal fluid of MSA patients and correlated with a greater distribution of AA genotype compared to healthy controls [185]. This genotype also manifested phenotypically with earlier onset of symptoms and greater progression of disease compared to non-AA genotypes.

Genetic polymorphisms in *IL-1R2*, *IL-1RA*, *IL-6*, *IL-10*, *TGF-β1*, *WNT3*, *HLA-DRB5* were not found to have significantly affected risk for developing MSA [163, 166, 186].

### Other Parkinson’s disease (PD)-related genes

There are overlapping mechanisms and common pathways in the pathogenesis of PD and MSA, given the shared clinical and histopathological features, coexistence of both diseases within the same pedigree [15], and higher rates of parkinsonism among 1st-degree relatives of MSA [14, 187]. The susceptibility risk of genetic polymorphisms known to be related to PD have thus been studied in MSA cohorts.

One study investigated dopamine metabolism-related gene polymorphisms known to alter PD risk of phenotype (*DDC* rs921451, *TH* rs6356, *COMT* rs4680, *MAOB* rs1799836, *DBH* rs1611115) [188]. *DDC* rs921451 minor allele “C” was associated with an increased risk of MSA, especially in male subjects, while haplotype analysis showed the “T-T” haplotype in *TH* rs6356 and *DDC* rs921451 risk alleles reduced the risk for MSA. *DDC* rs921451 T > C was associated with reduced expression or activity of *DDC*, an enzyme involved in dopamine and norepinephrine synthesis [189, 190]. This may consequently result in features of parkinsonism and autonomic dysfunction, which are cardinal features in MSA [191, 192].

Another study found a possible increased risk of MSA amongst female patients carrying the *NMD3* rs34016896 minor allele, which has been shown to correlate with nigral neuronal loss [193, 194], although it has not been shown to conclusively increase PD risk [195, 196].

Other genes and polymorphisms related to PD have been studied, but no associations have been found with MSA: *PARK2* (parkin) [197], *PINK1* [197], *SREBF1* (rs11868035) [198], *GPNMB* (rs156429) [199], *FBXO7* [200], *SLC1A2* (rs3794087) [201], *TMEM230* [202, 203], *TMEM106B* (rs1990622, rs3173615) [204], *VMAT2* (rs363371, rs363324) [204], *LINGO1* (rs11856808, rs9652490) [205], *LINGO2* (rs10968280, rs13362909, rs7033345) [205], *RAB7L1* (rs1572931) [206], *CHCHD2* [207], *DCTN1* [208] and *ATP13A2* [209].

### MSA genome-wide association studies

The only genome-wide association study conducted on MSA to date was published by Sailer et al. in 2016 [35]. It included subjects of European ancestry recruited from European and North American centers, with 291 out of 918 being pathologically-diagnosed cases. The study identified four loci of interest, *FBXO47*, *ELOVL7*, *EDN1*, and *MAPT*, but none surpassed the Bonferroni threshold for multiple testing. Notably, *COQ2* and *SNCA* specifically were not found to significantly modify MSA risk in this cohort. Wenick et al. investigated *ELOVL7* in a group of pathologically-diagnosed MSA patients, but could not identify any significant association [210].

### Gene-environment interactions and epigenetics

Analyzing the genetic risk profile of the disease without observing its interplay with environmental factors would be overly simplistic. There have been various studies reporting the association between MSA and various environmental risk factors (including organic solvents, plastic monomers/additives, pesticides, agricultural activities, metals and smoking) [187, 211–214], but data on gene-environment interactions are limited. A recent case–control study found differential risks between individuals who shared the same SNPs and had varied exposures to different environmental factors including smoking, alcohol, drinking well water and pesticide exposure [215]. Such-gene-environment interactions will be clinically relevant since different genotypes may accentuate or attenuate the impact of certain environmental factors on MSA risk. Similarly, epigenetics is becoming increasingly implicated in the development of MSA through regulation of gene expression. Recent genome-wide studies have shown altered DNA methylation profiles between MSA patients and healthy controls (including hypomethylation of *SNCA*), some of which are modified by various environmental exposures [216, 217]. More work needs to be done to delineate such complex relationships.

### Genetic model organisms

Animal models have been used to gain insight into the genetic basis of MSA [218]. For example, transgenic mouse models involving the overexpression of ɑ-synuclein in oligodendrocytes have been able to replicate MSA pathology and facilitate the understanding of GCI-linked neurodegeneration. However, this overexpression model may not fully recapitulate the processes seen in human model of MSA [219, 220]. Another transgenic mouse model showed that overexpression of ɑ1B-adrenergic receptors produced a MSA-like disorder with features of parkinsonism, autonomic dysfunction and ɑ-synuclein aggregation in oligodendrocytes [221]. However, the relevance and association of ɑ1B-adrenergic receptors to human MSA is unclear [222]. At present, there are no ideal MSA genetic models that have been developed and this should be a priority for investigators.

## Discussion and limitations

**Fig. 2:**
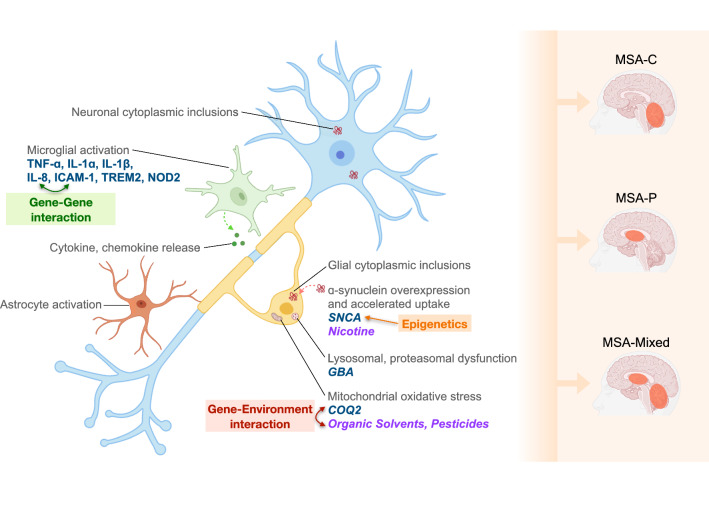
Pathophysiological mechanisms and underlying genetic aberrations that modulate MSA risk. The pathological hallmark of MSA is the presence of glial cytoplasmic inclusions. Our understanding of disease biology is inadequate, but purported mechanisms include ɑ-synuclein overexpression and accelerated uptake, oxidative stress, and microglial activation. These mechanisms are modulated by a range of genes, which have been shown to influence disease phenotype. This is further complicated by gene–gene interactions (e.g. *IL-8* and *ICAM-1*), gene-environmental interactions (e.g. between *COQ2* and organic solvents/pesticides) and epigenetics (e.g. DNA hypomethylation of *SNCA*). *MSA-C* Multiple System Atrophy (Cerebellar subtype), *MSA-P* Multiple System Atrophy (Parkinsonian subtype), *MSA-Mixed* Multiple System Atrophy (Mixed subtype)

We present a comprehensive review of genes associated with MSA (Table 1). We highlight the possible biological mechanisms, outline complex gene–gene and gene-environment interactions, and show how genetic variations influence disease phenotype (Fig. 2).

**Table 1:** Summary of genes associated with MSA

Gene	Gene product	Linked disorders	Mechanism	Evidence
*SNCA* (4q22.1)	ɑ-synuclein	PD (monogenic)	ɑ-synuclein is a major component of GCI, which is the main pathologic finding in MSA	*SNCA* SNPs rs11931074 [22, 23], rs3857059 [23], rs3822086 [24], rs3775444 [24] are associated with increased risk of MSA in Caucasian populations There is a possible role of *SNCA* CNVs, mainly gains, which correspond to increased ɑ-synuclein inclusions in cells [31–33]
*LRRK2* (12q12)	Leucine-rich repeat kinase 2	PD (monogenic)	Unknown	Some *LRRK2* polymorphisms (M2397T, G1624G, M1646T, N2081D, N551K, R1398H) may be protective against MSA [48]
*GBA* (1q21)	β-glucocerebrosidase	PD (monogenic)	Lysosomal dysfunction dysregulates ɑ-synuclein processing and induces aggregation	Several pathogenic *GBA* SNPs were associated with MSA, especially in North American cohorts, which may comprise a larger proportion of Ashkenazi Jewish patients [62, 64, 65]
*COQ2* (4q21.23)	Coenzyme Q2	–	CoQ deficiency results in mitochondrial oxidative stress with reduction in ATP synthesis	Reduction in *COQ2* expression with corresponding decrease in CoQ and ATP levels have been shown in both the brain tissue and plasma of MSA patients [76–78] *COQ2* V393A variant may increase MSA risk (especially MSA-C subtype) among East Asian populations [79, 85–87]
*MAPT* (17q21.31)	Microtubule associated protein tau	AD, PD, FTD, PSP, CBD, DLB	Tau confers neuronal microtubule stability, but aberrant deposition in neuronal or glial cells can result in neurodegenerative disorders	*MAPT* SNPs rs1052553, rs242557, rs3785883, rs8070723 may influence MSA risk [109, 110] Two risk haplotypes (H1x and H1J) and two protective haplotypes (H2 and H1E) were also found to modify MSA susceptibility, with the H2 haplotype showing a significant association for MSA-C and MSA-mixed subtypes only [110]
SCA-related - *ATXN1* *- ATXN2* *- TBP*	Includes: - Ataxin 1 (SCA-1) - Ataxin 2 (SCA-2) - TATA-box binding protein (SCA-17)	SCA	Unknown	Repeat expansions in SCA genes have been reported to increase risk for MSA, especially MSA-C. There is likely ethnic variation as *ATXN1* (SCA-1) and *ATXN2* (SCA-2) were implicated in an Italian population [121], but *TBP* (SCA-17) was involved in a Korean population [122]. There was also higher mean CAG repeat length in MSA patients compared to controls [121, 125, 126]
*C9orf72* (9p21.2)	chromosome 9 open reading frame 72	ALS, FTD	Unknown	An Italian study found *C9orf72* heterozygous mutations in the pathological range for two patients and intermediate/premutation range for four patients [150]
*RFC1* (4p14)	Replication Factor C Subunit 1	CANVAS	Unknown	One study discovered *RFC1* biallelic (AAGGG)exp and heterozygous (AAGGG)exp alleles in three and thirteen clinically-diagnosed MSA patients (n = 282) respectively, but this did not reach statistical significance [152]
*NOTCH2NLC* (1q21.2)	Notch Homolog 2 N-Terminal-Like Protein C	NIID	Unknown	Pathogenic *NOTCH2NLC* (GGC)exp was detected in 2.6% of clinically-diagnosed MSA patients [156]. These patients had longer disease duration, slower progression and ɑ-synuclein-negative skin biopsies, which suggests either MSA misdiagnosis or dual pathology
MSA GWAS	A diverse set of genes identified from GWAS	–	Various	A MSA GWAS identified four loci of interest, *FBXO47*, *ELOVL7*, *EDN1*, and *MAPT* [35] A study investigated *ELOVL7* in a group of pathologically-diagnosed MSA patients, but could not identify any significant association [210]

Despite the interesting observations from genetic studies in MSA, these must be interpreted with caution. To date, no large familial MSA pedigrees and monogenic forms have been identified. The genetic association studies reporting links with several genetic variants and loci do not determine an exact cause-effect relationship. Certain genes may contain innumerous disease-causing variants and haplotypes, thus preventing genome-wide association studies from detecting association signals from truly pathogenic genes. Most of these gene variants appear to confer a small or minimal effect size in the population, suggesting the possibility of other genetic determinants and contribution from environmental factors. Each gene may have an underlying set of gene regulators, or may in turn regulate other genes, hence adding further variables to an already convoluted genetic landscape. The sample sizes for most studies are small and do not have sufficient power to identify small differences. Furthermore, given that the phenotype of MSA is wide and varied, studies replying on solely clinical features assessed at a single time point may not be accurate. Most reported studies recruit clinically-diagnosed MSA patients based on the consensus statement proposed by Gilman et al. [5], as opposed to neuropathological criteria. One estimate places the clinical diagnostic accuracy of MSA at only 62% [223]. Thus, these studies may contain a sizeable minority of patients who do not actually have MSA, but rather a MSA-mimic such as other Parkinson-Plus syndromes with differing genetic susceptibility.

## Conclusion and future directions

Although MSA is largely sporadic, genetic studies have allowed us to understand potential genetic factors that underpin the disease. Current studies have suggested possible associations between MSA risk and a wide range of gene mutations and polymorphisms. These genes include those linked to other common neurodegenerative conditions and those which are known to play a major functional role on oxidative stress, neuroinflammation, and protein degradation. However, thus far no monogenic forms of MSA have been identified. Multinational and multicenter studies with longitudinal follow up data will be helpful in identifying rare gene variants with small effect sizes and delineating heterogeneity between various age, sex and ethnic subgroups. In addition, large scale epidemiologic cohorts will also facilitate the identification of gene–gene and gene-environmental interactions. Functional studies in both animal and human models of the various identified genetic variants/mutations can identify novel pathophysiologic clues which may lead to development of disease-modifying therapeutic targets [224–226].

## Data Availability

Not applicable.

## Abbreviations

AD: Alzheimer’s disease
ALS: Amyotrophic lateral sclerosis
CBD: Corticobasal degeneration
CNV: Copy number variation
DLB: Dementia with lewy bodies
FTD: Frontotemporal dementia
GCI: Glial cytoplasmic inclusions
MSA: Multiple system atrophy
NIID: Neuronal intranuclear inclusion disease
PD: Parkinson’s disease
PSP: Progressive supranuclear palsy
SCA: Spinocerebellar ataxia
SNP: Single nucleotide polymorphism
